# Association of DNA Copy Number Aberrations with Organ-Specific Metastasis in Luminal B HER2-Negative Breast Cancer

**DOI:** 10.3390/diseases14100352

**Published:** 2026-09-23

**Authors:** Marina K. Ibragimova, Matvey M. Tsyganov, Arina K. Shagabudinova, Irina A. Tsydenova, Maxim A. Morozovsky, Nikolai V. Litviakov

**Affiliations:** 1 Cancer Research Institute, Tomsk National Research Medical Center, Russian Academy of Sciences, 5 Kooperativny Lane, Tomsk 634050, Russia; tsyganovmm@yandex.ru (M.M.T.); arisha.shagabudinova@mail.ru (A.K.S.); tsydenova422@gmail.com (I.A.T.); morozovskiy0m@gmail.com (M.A.M.); nvlitv72@yandex.ru (N.V.L.); 2 Biochemistry and Molecular Biology Division, Siberian State Medical University, Moskovsky Trakt 2, Tomsk 634050, Russia; 3 Institute of Biology, National Research Tomsk State University, 36 Lenin Ave., Tomsk 634050, Russia

**Keywords:** breast cancer, neoadjuvant chemotherapy, microarray analysis, hematogenous metastasis, organ specificity

## Abstract

Breast cancer (BC) exhibits considerable metastatic heterogeneity and distinct patterns of organotropism, which contribute to differences in patient outcomes and therapeutic responses. However, the molecular mechanisms underlying this organ specificity remain incompletely understood. Objective: To investigate the genetic landscape of primary breast tumor, surgical specimens, and metastatic lesions and to identify copy number aberration (CNA) regions associated with organ-specific metastatic-site patterns in breast cancer. Materials and methods: The study included paired pretreatment and post-treatment surgical specimens from 50 patients with BC (T1–4N0–3M0) and hematogenous metastases. In addition, three samples (biopsy, surgical specimen, and metastatic lesion) were obtained from four patients with primary metastatic BC (with metastasis to the bones, lungs, liver, and brain). Microarray analysis was performed using high-density CytoScan^TM^ HD Array DNA chips (Affymetrix (USA)). Results: The following CNA regions were associated with organ-specific metastatic-site patterns: bone metastasis was associated with a higher frequency of amplifications in the 14q32.33 region (*p* = 0.0144); liver metastasis was associated with a higher frequency of amplifications in the 17q23.2 region (*p* = 0.0042, confirmed by validation on an independent cohort); lung metastasis was associated with a higher frequency of deletions in the 2p13.1 and 19p13.12 regions (*p* = 0.0449); and brain metastasis was associated with a higher frequency of deletions in the 19q12 region (*p* = 0.0522) and in the 20q11.22 region (*p* = 0.0089, confirmed by validation in an independent cohort). Conclusions: Our findings identify CNA regions associated with organ-specific metastatic-site patterns in breast cancer patients.

## 1. Introduction

Metastasis represents a critical stage of tumor progression and involves the dissemination of malignant cells from the primary tumor to distant organs. Hematogenous metastasis is a multistep process commonly conceptualized as a sequence of events that includes local invasion and detachment from the primary tumor, intravasation and survival in the bloodstream, extravasation, and colonization of distant sites. This process involves the clonal evolution of tumor cells in vivo and dynamic interactions with the tumor microenvironment, underscoring the need for a comprehensive approach to understanding metastatic potential [1].

Metastasis is responsible for more than 90% of cancer-related deaths, frequently as a result of disruption of vital organ function [2]. In breast cancer, the incidence of metastasis varies considerably according to clinical and morphological characteristics, reaching 50–75% in the presence of multiple unfavorable prognostic factors [3]. Numerous clinical studies have demonstrated that tumor metastasis exhibits organ specificity, also known as organotropism, whereby different tumor types preferentially metastasize to specific organs. For example, in patients with breast cancer, bone metastases occur in 60–75% of cases, lung metastases in 32–37%, liver metastases in 32–35%, and brain metastases in 10% [4,5]. The frequency of gastrointestinal tract metastases in breast cancer ranges from 4% to 8% [6], while adrenal gland metastases are rare [7]. Ovarian metastasis remains a controversial aspect of organ-specific dissemination in breast cancer, with reported incidences ranging from 3% to 47% [8,9].

Organ-specific metastasis is influenced by numerous factors, particularly the clinical, pathological, and molecular characteristics of the primary tumor [10,11]. Indeed, different cancer types and molecular subtypes exhibit distinct patterns of metastatic dissemination [12,13,14]. For example, triple-negative breast cancer (TNBC), which accounts for approximately 10–15% of all cases, is particularly prone to visceral metastasis (with lung metastasis rates reaching 40%) [15], whereas the highest rates of bone metastasis (up to 65–70%) are observed in patients with luminal breast cancer [3].

The prognostic significance of metastatic patterns remains controversial. Some studies suggest that the number of metastatic foci is an independent predictor of poor outcome, whereas others indicate that molecular subtype may play a decisive role regardless of the extent of metastasis [16]. For example, the first population-based study to systematically characterize 15 combinations of metastatic sites and their association with survival demonstrated that metastases involving multiple organs, particularly combinations involving the brain (e.g., brain + liver + lungs), were associated with the poorest prognosis, with a median overall survival of 4.0 months. Young age (≤40 years), high histological grade (grade III), and tumor location in the axillary region or unspecified sites were independently associated with an increased risk of metastasis [17].

However, no diagnostic tools are currently available to predict the site of metastasis or to facilitate monitoring and the development of organ-specific interventions. Studies investigating the characteristics of distant metastases and potential predictive markers remain limited. For example, in a study by Jamie R. Kutasovic et al., primary and metastatic tumors from 54 patients with breast cancer and gynecological metastases (ovary, fallopian tube, omentum/peritoneum) were analyzed using DNA copy number profiling and targeted sequencing. The authors found that primary tumors frequently expressed estrogen receptor cofactors (*GATA3*, *FOXA1*) and harbored amplifications at 8p12, 8q24, and 11q13. Regarding phenotypic conversion, estrogen receptor status was generally preserved in metastases, while *FOXA1* expression was increased, and expression of progesterone receptor, androgen receptor, and *GATA3* was decreased [18].

Additional studies have reported that the transcription factor SP7 promotes bone metastasis in breast cancer by upregulating genes involved in the metastatic cascade [19]. Furthermore, TGF β, which may initially act as a tumor suppressor, subsequently promotes breast cancer metastasis to bone by enhancing epithelial–mesenchymal transition, angiogenesis, and immunosuppression. Claudin-2 has been implicated in liver metastasis [20], and HER2, through its effects on cellular proliferation and survival, contributes to brain metastasis and may be associated with tropism for the central nervous system [21].

Elucidating the mechanisms of organotropism in tumor metastasis is critical for the identification of novel therapeutic targets and strategies and represents an important frontier in precision medicine. Therefore, the present study investigated the genetic landscape of primary breast tumors, surgical specimens, and metastatic lesions to identify CNA regions associated with organ-specific metastatic-site patterns in luminal B HER2-negative breast cancer.

## 2. Materials and Methods

Patients and treatment. The retrospective study included 50 patients aged 24–68 years (mean age, 47.1 ± 0.3 years; mean ± SE) with a morphologically confirmed diagnosis of luminal B HER2-negative breast cancer, stage T1-4N0-3M0 (IIA–IIIC). Primary breast lesions were assessed by mammography and ultrasonography. These patients presented with various stages of the disease. During the follow-up period (mean duration: 64 months; see Table 1), these patients developed hematogenous metastases. As the study utilized patient biopsy material obtained prior to treatment, the disease stage was assessed at the time of initial presentation.

In accordance with the Consensus Conference on Neoadjuvant Chemotherapy in Carcinoma of the Breast (26–28 April 2003, Philadelphia, PA, USA), patients received 4–8 cycles of neoadjuvant chemotherapy (NAC) according to one of the following regimens: FAC (fluorouracil, doxorubicin, cyclophosphamide), AC (doxorubicin, cyclophosphamide), CAX (cyclophosphamide, doxorubicin, capecitabine), CP (cyclophosphamide, cisplatin), ACT (doxorubicin, cyclophosphamide, docetaxel), AT (doxorubicin, docetaxel), and taxotere monotherapy. The efficacy of preoperative chemotherapy was assessed according to the criteria established by the World Health Organization (WHO) and the International Union Against Cancer. Tumor response was evaluated by ultrasound and/or mammography before treatment, after two cycles of NAC, and before surgery. The following responses were recorded: complete regression (100% tumor reduction), partial regression (more than 50% tumor volume reduction), stable disease (less than 50% volume reduction or no more than 25% increase), and progressive disease (more than 25% tumor volume increase). All cases were confirmed morphologically. All patients subsequently underwent surgery, which included radical or subcutaneous mastectomy, radical resection, sectoral resection with axillary lymphadenectomy, or other breast-conserving procedures. Patients who achieved partial regression, stable disease, or progressive disease after NAC were included in the study. Patients who achieved complete regression were excluded due to the lack of tumor material after NAC. The main clinical and morphological parameters of the patients included in the study are summarized in Table 1.

The study material consisted of paired samples, including pretreatment biopsy specimens and postsurgical specimens obtained from each patient. Tumor biopsy specimens were collected before treatment using core needle biopsy under ultrasound guidance.

In addition, three samples (biopsy specimen, surgical specimen, and metastatic lesion) were obtained from four patients with primary metastatic breast cancer (aged 24–68 years (mean age 47.1 ± 0.3 years; mean ± SE) with a morphologically confirmed diagnosis of luminal B HER2-negative breast cancer, stage T3-4N2-3M1 (IV), with metastases to the bones, lungs, liver, and brain. All patients had confirmed hematogenous metastases at initial presentation.

The main clinical and morphological parameters of the four patients with primary metastatic breast cancer included in the study are summarized in Table 2.

DNA was extracted from paired tumor and metastasis tissue samples using the QIAamp DNA mini Kit (Qiagen, Hilden, Germany) in accordance with the manufacturer’s instructions.

Microarray analysis was performed using high-density CytoScan^TM^ HD Array microarrays (Affymetrix, Santa Clara, CA, USA). Sample preparation, hybridization, and scanning were carried out according to the manufacturer’s protocol on an Affymetrix GeneChip^®^ Scanner 3000 7G (Affymetrix, USA). The results were analyzed using Chromosome Analysis Suite 4.0 software (Affymetrix, USA) (https://documents.thermofisher.com/TFS-Assets/LSG/manuals/ChAS_Manual.pdf) (accessed on 20 March 2026). The following thresholds were used for CNA analysis: gain (Marker Count–50, Size (kbp)–400); loss (Marker Count–50, Size (kbp)–400); SNPQC > 16, MAPD < 0.23; Waviness SD < 0.1 (Minimum log2 ratio found in the chromosome); The analysis included 851 chromosomal cytobands and 3000 segments.

Statistical analysis was performed using the “Statistica 13.0” software package (StatSoft Inc., Stanford Research Park Palo Alto, CA, USA). Frequencies of categorical variables were compared using Fisher’s exact two-tailed test.

A permutation test was used to test statistical hypotheses and determine whether the observed differences between two or more groups were unlikely to have arisen by chance. A *p*-value of less than 0.05 indicates that a difference in the observed magnitude occurred in fewer than 5% of random permutations, providing evidence against the null hypothesis of no difference between the groups.

Test characteristics were as follows: test type–paired groups (before/after) in the Section 3.2, statistic–difference in means, method–exact, reference–0.05, CI level–95%.

To control the error rate when comparing frequencies across two or more groups, a multiple hypothesis testing correction (Bonferroni correction) was applied. Details of the statistical analysis (use of a correction for multiple comparisons): a correction for multiple comparisons was applied using the formula a^new^ = 0.05/m (where a^new^ is the new critical significance level and m is the number of tests performed). Calculations accounted for pairwise comparisons and the frequencies of amplifications and deletions.

For multiple hypothesis testing correction, the number of subgroup comparisons was taken into account, whereas the number of loci analyzed in the microarray analysis was not. This limitation may have reduced the statistical power of the analysis and should be considered when interpreting the findings.

## 3. Results

### 3.1. Characteristics of the CNA Landscape in Pretreatment Tumors According to the Organ Specificity of Distant Metastasis

The association between the presence of distant metastasis (single-organ versus multiple-organ metastasis) and the frequency of CNA in pretreatment tumor samples was investigated (Figure 1a). Patients were stratified into two groups: those with metastasis to a single organ (n = 30) and those with metastasis to two or more organs (n = 20).

The clinical characteristics of organ-specific distant metastasis in the study cohort are summarized in Table 3.

**Table 3:** Characteristics of distant metastasis sites in patients with BC.

General Characteristics		
Single-organ metastasis		30 (60.0%)
Multiple-organ metastasis		20 (40.0%)
Spread		
One organ		Total
Bones	6 (12.0%)	21 (42.0%)
Lungs	9 (18.0%)	19 (38.0%)
Liver	12 (24.0%)	16 (32.0%)
Brain	1 (2.0%)	8 (16.0%)
Ovary	0 (0.0%)	1 (2.0%)
Mediastinum	0 (0.0%)	1 (2.0%)

Comparison of CNA frequencies between the two patient groups revealed that patients with metastases to two or more organs showed higher frequencies of amplification at 6p21.1 and 7p14.1. Specifically, amplifications in these regions were detected in 30% of patients with multiple-organ metastases, whereas no such amplifications were detected in the single-organ metastasis group (*p* = 0.008). Similarly, metastases to two or more organs were associated with a higher frequency of deletions at 15q22.1, which were observed in 23.5% of patients in this group, with no deletions detected in the single-organ metastasis group (*p* = 0.016).

Conversely, single-organ metastasis was associated with a higher frequency of amplification at 5q35.2. Amplification of this region was detected in 25% of patients with single-organ metastasis, whereas no amplification was detected in patients with multiple-organ metastases (*p* = 0.025).

Permutation test results comparing the frequencies of amplifications in the 6p21.1, 7p14.1, and 5q35.2 regions and deletion in the 15q22.1 region between groups with metastasis to a single organ and to two or more organs: *p* = 0.042296, permutation count = 330, observed (effect) = −1.932773 × 10^12^, Cohen’s d = −0.969239. The observed difference between the groups was statistically significant.

After applying a multiple hypothesis testing correction (Bonferroni correction), statistically significant differences in amplification frequencies at 6p21.1 and 7p14.1 remained in patients with metastases to two or more organs (*p* = 0.008).

Further comparison of CNA frequencies in pretreatment tumors according to the specific site of distant metastasis identified several potential CNA regions associated with organ-specific metastatic-site patterns (Supplementary Table S1).

A simultaneous comparison of SNA across four groups (stratified by the target organ of metastasis) yielded the following data:

Bone metastasis was associated with a higher frequency of amplification at 14q32.33, detected in 38.1% of patients with bone metastases, whereas no amplification was detected in patients with brain metastases (*p* = 0.0144).

Liver metastasis was associated with a higher frequency of amplification at 17q23.2, identified in 37.5% of patients with liver metastases, whereas no amplification was detected in the bone metastasis group (*p* = 0.0042).

Lung metastasis was associated with higher deletion frequencies at 2p13.1 and 19p13.12. Deletions in these regions were observed in 21.0% of patients with lung metastases and were absent in the bone metastasis group (*p* = 0.0449).

Brain metastasis was associated with a higher deletion frequency at 19q12, detected in 25.0% of patients with brain metastases, whereas no such deletions were detected in the lung metastasis group (*p* = 0.0522). Brain metastasis was also associated with a higher frequency of deletion at 20q11.22, detected in 37.5% of patients with brain metastases, whereas no such deletions were observed in the liver metastasis group (*p* = 0.0089).

Permutation test results comparing the frequencies of amplifications in the 14q32.33, and 17q23.2 regions and deletions in the 2p13.1, 19p13.12, 19q12 and 20q11.2 regions between groups with bone, liver, lung and brain metastasis: *p* = 0.048338, permutation count = 330, observed (effect) = −5.857724, Cohen’s d = −0.451224. The observed difference between the groups was statistically significant.

The distribution of CNA frequencies in patient groups stratified by organ-specific distant metastasis is shown in Figure 2a.

After application of the Bonferroni correction, statistically significant amplification frequencies at 17q23.2 (*p* = 0.0042) were identified in cases of liver metastasis (n = 16) (Figure 1b), while statistically significant deletion frequencies at 20q11.22 (*p* = 0.0089) were associated with brain metastasis (n = 8), with adjustment for the error rate in comparisons across two or more groups.

### 3.2. Characteristics of the CNA Landscape in Tumors Before and After NAC in Relation to Organ-Specific Distant Metastasis

We next analyzed the association between organ-specific distant metastasis and changes in CNA frequency before and after NAC. Patients were stratified into four groups based on metastatic site: bone (n = 21), lung (n = 19), liver (n = 16), and brain (n = 8).

In the bone metastasis group, comparison of pretreatment and post-NAC tumor samples revealed a statistically significant increase in the frequency of amplification at 17q23.1 locus (from 0% to 38.1%; *p* = 0.00172). A significant increase in the deletion frequency was also observed at 2p12 and 3p11.1 (from 0% and 19.0%; *p* = 0.036).

In the lung metastasis group, analysis of changes in tumor CNA frequencies following NAC revealed a significant increase in amplification frequency at 2p16.3 and 6q15 (from 0% to 26.3%; *p* = 0.017). Conversely, the deletion frequencies at Xq11.2 and Xq21.31 decreased significantly after treatment, from 21.1% to 0% (*p* = 0.046).

In the liver metastasis group, a significant increase in amplification frequency at the 7p12.3 locus was observed, from 0% to 37.6% (*p* = 0.007). Similarly, the frequency of deletion at the 1p12 locus increased significantly from 0% to 25.0% (*p* = 0.033). In contrast, significant decreases were observed after NAC in the frequency of amplification at 2p25.1, from 25.0% to 0% (*p* = 0.033), and deletion at 10q26.3, from 25.0% to 0% (*p* = 0.033).

In the brain metastasis group, amplification frequency was observed at 17q24.2 (from 37.5% to 0%; *p* = 0.05). This difference was not statistically significant.

Permutation test results comparing the frequencies of amplifications in the 17q23.1, 2p16.3, 6q15, 7p12.3, 2p25.1 and 17q24.2 regions and deletions in the 2p12, 3p11.1, Xq11.2, Xq21.31, 1p12 and 10q26.3 regions between groups with bone, liver, lung and brain metastasis before and after neoadjuvant chemotherapy: *p* = 0.046465, permutation count = 330, observed (effect) = −6, Cohen’s d = −0.134867. The observed difference between the groups was statistically significant.

After applying a multiple hypothesis testing correction and controlling for the error rate in frequency comparisons across two or more groups, a statistically significant increase in the frequency of amplifications at 17q23.1 from 0% to 38.1% (before/after neoadjuvant chemotherapy) was observed following preoperative chemotherapy in patients with bone metastases (*p* = 0.00172). In addition, a statistically significant increase in the amplification frequency at locus 7p12.3 from 0% to 37.6% (before/after NAC) was identified following preoperative chemotherapy for patients with liver metastases (*p* = 0.007).

### 3.3. CNA Landscape Characteristics of Paired Primary Tumor and Metastatic Samples in Patients with Primary Metastatic Breast Cancer (Illustrative Case Examples from Our Cohort)

To evaluate the identified CNA regions associated with organ-specific metastatic-site patterns, we analyzed the status of the relevant genomic regions in paired samples (biopsy specimen, surgical specimen, and metastatic lesion), taking into account the target organ of distant metastasis (Table 4).

A qualitative analysis was performed. Specifically, the status of CNA regions associated with organ-specific metastatic-site patterns identified in the overall study cohort was compared with the status of the corresponding loci in four patients with primary metastatic breast cancer. The data obtained were compared with the status of the identified loci in four primary metastatic cases (each including the following samples: pretreatment biopsy material, surgical material obtained after neoadjuvant chemotherapy and a metastasis lesion sample from the corresponding distant organ) representing different patterns of distant metastasis (bone, lung, liver, and brain metastases).

All identified CNA markers associated with organ-specific metastatic-site patterns were analyzed; markers that remained statistically significant after Bonferroni correction are highlighted in bold.

Thus, the status of CNA loci identified as potential CNA regions associated with organ-specific metastatic-site patterns in the overall patient cohort was compared with the status of these loci in four cases of primary metastatic breast cancer. Using samples from patients with disseminated breast cancer as examples, concordance of potential organ-specific CNA regions was observed for bone metastasis (14q32.33 amplification), lung metastasis (2p13.1 deletion), liver metastasis (17q23.2 amplification), and brain metastasis (19q12 and 20q11.22 deletions).

According to the GeneCards database (https://www.genecards.org/), chromosomal locus 14q32.33 is a biologically significant genomic region that plays a central role in several key intracellular signaling pathways, primarily due to the genes located within this region. It contains important regulators of cell proliferation and apoptosis, in particular the *PACS2*, *AKT1*, and *JAG2* genes. The 2p13.1 locus contains several important genes involved in nervous system function, smooth muscle contraction, and intracellular transport, including *DCTN1*, *ACTG2*, and *HTRA2*. Chromosomal region 19p13.12 is a critically important area of the genome that is actively involved in transcriptional regulation and apoptosis. It contains several key genes, the most well-known of which are *NOTCH3*, *PKN1*, and *BRD4*, which are frequently implicated in cancer development and progression. Chromosomal region 17q23.2 contains several genes, the most important of which are the transcription factor genes *TBX2* and *TBX4*. The 19q12 locus is a genomic region critically involved in cell cycle regulation and contains the genes *CCNE1* and *URI1*. The 20q11.22 region contains several genes that are crucial for cell signaling, metabolism, and embryonic development. Aberrations within this region contribute to both oncogenesis and stem cell evolution.

Table 5 summarizes the potential involvement of the identified CNA regions associated with organ-specific metastatic-site patterns in cellular signaling pathways based on information from the GeneCards database. (https://www.genecards.org/).

Thus, it has been shown that all of the above-mentioned loci contain genes that directly or indirectly play an important role in cell cycle regulation (proliferation, transcription, apoptosis), acting as transcription factors, which confirms the importance of determining the status of these loci when considering cancer progression.

We subsequently performed a similar qualitative analysis to compare the status of CNAs associated with organ-specific metastasis before and after chemotherapy, as determined in the overall patient cohort (Table 6). The obtained data were also correlated with the status of the identified loci in four primary metastatic cases (each including the following samples: pretreatment biopsy material, surgical material obtained after NAC, and a metastatic lesion specimen from the corresponding distant organ) representing different patterns of distant metastasis (bone, lung, liver, and brain metastases).

At this stage, all CNAs associated with organ-specific metastasis in tumors under NAC exposure were analyzed; markers found to be statistically significant after Bonferroni correction are highlighted in bold.

Thus, using disseminated breast cancer samples as examples, alterations in CNA regions specific to metastasis under NAC were correlated for bone metastasis (17q23.1 amplification, 3p11.1 deletion), lung metastasis (2p16.3 and 6q15 amplifications); and liver metastasis (7p12.3 amplifications and 1p12 deletion). In the presented analysis, specificity was defined as concordance of CNA status in two or more corresponding samples for each case (B + S/B + M/S + M).

### 3.4. Validation of the Results in an Independent Cohort

Using data from the publicly available cBioPortal database (https://www.cbioportal.org/), the frequency of potential CNA regions associated with organ-specific metastatic-site patterns identified in the present study was analyzed. In total, 1605 breast cancer patients with available CNA status data and information on the site of distant metastasis localization were included in the analysis.

The analysis included two CNA regions associated with organ-specific metastatic-site patterns, which underwent adjustment for the error rate in comparisons across two or more groups. According to the presented results, after applying a multiple hypothesis testing correction, statistically significant amplification frequencies at 17q23.2 (*p* = 0.0042) were identified in cases of liver metastasis (Figure 1b), while statistically significant deletion frequency at 20q11.22 (*p* = 0.0089) was associated with brain metastasis.

Initially, the frequency of amplification at 17q23.2 was analyzed in cases of liver metastasis (n = 490) and compared with the frequency of amplification in the same region in metastases to other distant organs (bones, lungs, and brain) (n = 1284) in patients with breast cancer.

It was demonstrated that amplification of this region occurred significantly more frequently in liver metastasis cases (56.9%, 279 out of 490 cases) than in metastases to other distant organs (25.0%, 321 out of 1284 cases) (OR = 3.97(95% CI 3.19 to 4.94), *p* = 5.64 × 10^−11^ (after applying a multiple hypothesis testing correction and controlling for the error rate in frequency comparisons across two or more groups)), which is consistent with the findings obtained in the present study. The frequency of amplifications at 17q23.2 in liver metastases and metastases to other distant organs according to the publicly available cBioPortal database is presented in Figure 3.

The frequency of deletion at 20q11.22 was also analyzed in patients with brain metastasis (n = 96) and compared with that in patients with metastases to other distant organs, including bone, lung, and liver (n = 1509).

Deletion of this region occurred significantly more frequently in patients with brain metastasis (20.8%, 20 out of 96 cases) than in patients with metastases to other distant organs (4.0%, 51 out of 1.283 cases) (OR = 6.36 (95% CI 3.61 to 11.2), *p* < 0.0000001), consistent with the findings of the present study. The frequency of deletion at 20q11.22 in brain metastases and metastases to other distant, based on data from the publicly available cBioPortal database, is shown in Figure 4.

## 4. Discussion

We found that metastasis to two or more organs was associated with higher amplification frequencies at 6p21.1 and 7p14.1. Specifically, these amplifications were detected in 30% of patients with multiple-organ metastases, whereas they were completely absent in the group with single-organ metastases (*p* = 0.008).

The literature on CNA features associated with multiple distant metastases remains limited. However, a recent study using comparative genomic hybridization and transcriptomic microarray analysis in triple-negative breast cancer reported that multiple-organ metastases were characterized by activation of signaling pathways involving *HSPA1L*, *HSPA1B*, *HSPA1A*, and *CSNK2B* (mapped to chromosome 6p21.33) [22].

Comparison of pretreatment CNA frequencies stratified by organ-specific distant metastasis revealed several potential CNA regions associated with organ-specific metastatic-site patterns.

Bone metastasis was associated with a higher frequency of amplification at 14q32.33, which was detected in 38.1% of patients with bone metastases but was absent in the brain metastasis group (*p* = 0.0144).

A previous transcriptomic study reported metastatic-site-specific expression patterns involving *ADSS1*, *AKT1*, and *KLC1* in brain metastases. These genes have been mapped to the broader 14q32 region and therefore provide functional context for the 14q32.33 locus identified in our analysis. However, the different metastatic site and molecular endpoint examined in that study do not allow a direct comparison with our CNA finding [22].

Lung metastasis was associated with a higher frequency of deletion at 2p13.1, which was detected in 21.0% of patients with lung metastases but was absent in the bone metastasis group (*p* = 0.0449).

The *LOXL3* gene is located within the 2p13.1 region. This oncoprotein has previously been implicated in promoting tumor development. These observations provide functional context for the genomic region identified in our analysis. However, although *LOXL3* is expressed in highly malignant invasive breast cancer, no correlation has been observed between LOXL3 expression and tumor size, mitotic index, or lymph node status [23]. Further studies are needed to clarify the relationship between *LOXL3* and breast cancer, as well as the underlying mechanisms involved.

It has also been shown that brain metastasis was observed with a higher frequency of deletion at 19q12, identified in 25.0% of patients with brain metastases, whereas no such deletions were found in the lung metastasis group (*p* = 0.0522).

The *CCNE1* gene, located on chromosome 19q12, is frequently amplified in various cancers [24]. However, data regarding the association between *CCNE1* amplification and poor prognosis in breast cancer are conflicting. In the Metabric cohort, *CCNE1* amplification was not associated with overall survival in ER-positive breast cancer [25]. In contrast, in a cohort of 54 cases of triple-negative breast cancer lacking *BRCA1/BRCA2* mutations, *CCNE1* amplification detected by targeted exome sequencing was associated with poor recurrence-free survival [26]. Further large-scale studies will be essential to fully elucidate the relationship between CNA involving *CCNE1* and prognosis in breast cancer.

Genomic and transcriptomic analyses have also identified aberrations that may help distinguish metastatic teratomas from primary testicular germ cell tumors. In particular, five loci (10q26.3, 16p11.2, 19p13.2, 19q12, and 22q12) exhibited recurrent gene loss events in metastatic teratomas compared to primary tumors [27].

Similarly, brain metastasis was associated with a higher frequency of deletion at 20q11.22, detected in 37.5% of patients with brain metastases, whereas no such deletions were observed in the liver metastasis group (*p* = 0.0089).

A whole-genome SNP array analysis of paired samples from patients with colorectal cancer and lung or brain metastases identified an increased frequency of heterozygosity loss at 20q11.21-q12 in brain metastases [28].

We next investigated changes in the CNA landscape following NAC using an observational comparison of CNA frequencies before and after chemotherapy in tumors stratified by the site of subsequent distant metastasis.

In the bone metastasis group, a statistically significant increase was observed in the frequency of amplification at 17q23.1 (*p* = 0.00172) and deletion at 2p12 and 3p11.1 (*p* = 0.036) after NAC.

According to the literature, epigenetic profiling for prognostic stratification and personalized therapy of breast cancer has identified five most common amplification and deletion regions, especially in genes located at 17q23.1 and 15q13.1 [29].

In the lung metastasis group, we observed a significant increase in amplification frequency at 2p16.3 and 6q15 (*p* = 0.017) and a significant decrease in deletion frequency at Xq11.2 and Xq21.31 (*p* = 0.046) following NAC.

The *PPP1R21* gene is located at 2p16.3 and has previously been studied in the context of breast cancer research, during which an association between expression and breast cancer was discussed. According to Cebria-Costa et al., *PPP1R21* expression increases in triple-negative breast cancer cell lines following *LOXL2* knockdown [30]. These findings provide functional context for the 2p16.3 region identified in our study.

Heterozygous deletions involving 6q15 have previously been reported in 18% of breast cancer cases and were significantly associated with advanced tumor stage and high malignancy (*p* < 0.0001) [31]. This previous observation provides contextual support for the potential relevance of the 6q15 region.

In the liver metastasis group, following NAC, a significant increase in amplification frequency at 7p12.3 (*p* = 0.007) and deletion frequency at 1p12 (*p* = 0.033) was found. At the same time, significant decreases were observed in amplification frequency at 2p25 (*p* = 0.033) and deletion frequency at 10q26.3 (*p* = 0.033) following treatment.

Genomic and transcriptomic profiling of inflammatory breast cancer by Zhou et al. identified 10 CNV regions significantly enriched in inflammatory breast cancer compared to non-inflammatory subtypes, including 7p12.3, 7q11.21, 7q36.3, 8p12, 9p24.1, 11p15.5, 11q14.2, 14q32.11, 14q32.33, and 16p13.3 (*p* < 0.05) [32].

The *ITGB1BP1* gene, located at 2p25.1, has been described as a transcriptional target of the CD44 signaling pathway, contributing to breast cancer cell invasion and metastasis [33].

Finally, in the brain metastasis group, a decrease in amplification frequency was observed at 17q24.2 following NAC (*p* = 0.05).

According to the literature, analysis of PPAR-associated long non-coding RNAs in breast cancer has revealed strong correlations between *ABCA9-AS1* (located at 17q24.2) and *KCNIP2-AS1* (correlation coefficient = 0.85) in tumor samples compared to normal tissue. These findings suggest that long non-coding RNAs may contribute to the pathogenesis of breast cancer [34].

## 5. Conclusions

Metastatic progression represents a major therapeutic challenge, as substantial tumor heterogeneity, both between patients and within individual tumors, remains a significant obstacle in the search for a rational therapeutic approach.

Over the past decade, advances in breast cancer genomics have substantially expanded our understanding of intratumoral heterogeneity, which is increasingly recognized as a major driver of cancer progression. Nevertheless, our understanding of metastatic breast cancer remains comparatively limited relative to that of primary breast cancer [35].

In the present study, we investigated the genetic landscape of primary breast tumors, post-treatment surgical specimens, and metastatic lesions to identify potential CNA regions associated with organ-specific metastatic-site patterns in luminal B/HER2-negative breast cancer.

Our results identified CNA regions associated with organ-specific metastatic-site patterns (with two of the six identified regions confirmed in an independent cohort) in breast cancer patients. We also identified alterations in specific CNA regions associated with the metastatic site following preoperative chemotherapy.

Elucidating the organ-specific characteristics of metastatic potential in breast cancer is critical for the development of novel diagnostic and prognostic strategies. However, additional studies are warranted to validate the identified markers (CNA regions associated with organ-specific metastatic-site patterns) for future clinical application.

### Limitations of the Study

(1) The primary limitation of this study is the relatively small sample size (n = 50), which was restricted to a single molecular subtype and included patients treated with different neoadjuvant chemotherapy regimens. This may have reduced the statistical power of the analysis and limited the generalizability of the findings to a broader population.
(2) The study design resulted in overlap among the metastatic groups because a single patient could have metastases involving multiple organs. The bone, lung, liver, and brain metastasis groups were therefore analyzed separately. This overlap may have reduced the statistical power of the analysis and limited the generalizability of the results.
(3) The findings may not be generalizable to breast cancer subtypes other than luminal B/HER2-negative tumors.
(4) In the multiple-comparison correction, the number of subgroup comparisons was taken into account, whereas the number of loci analyzed in the microarray analysis was not. This limitation may have reduced the statistical power of the analysis and should be considered when interpreting the findings.
(5) Validation of the results on an independent cohort (cBioPortal database) utilized data from breast cancer patients for whom the molecular subtype was not specified.

## Figures and Tables

**Figure 1:**
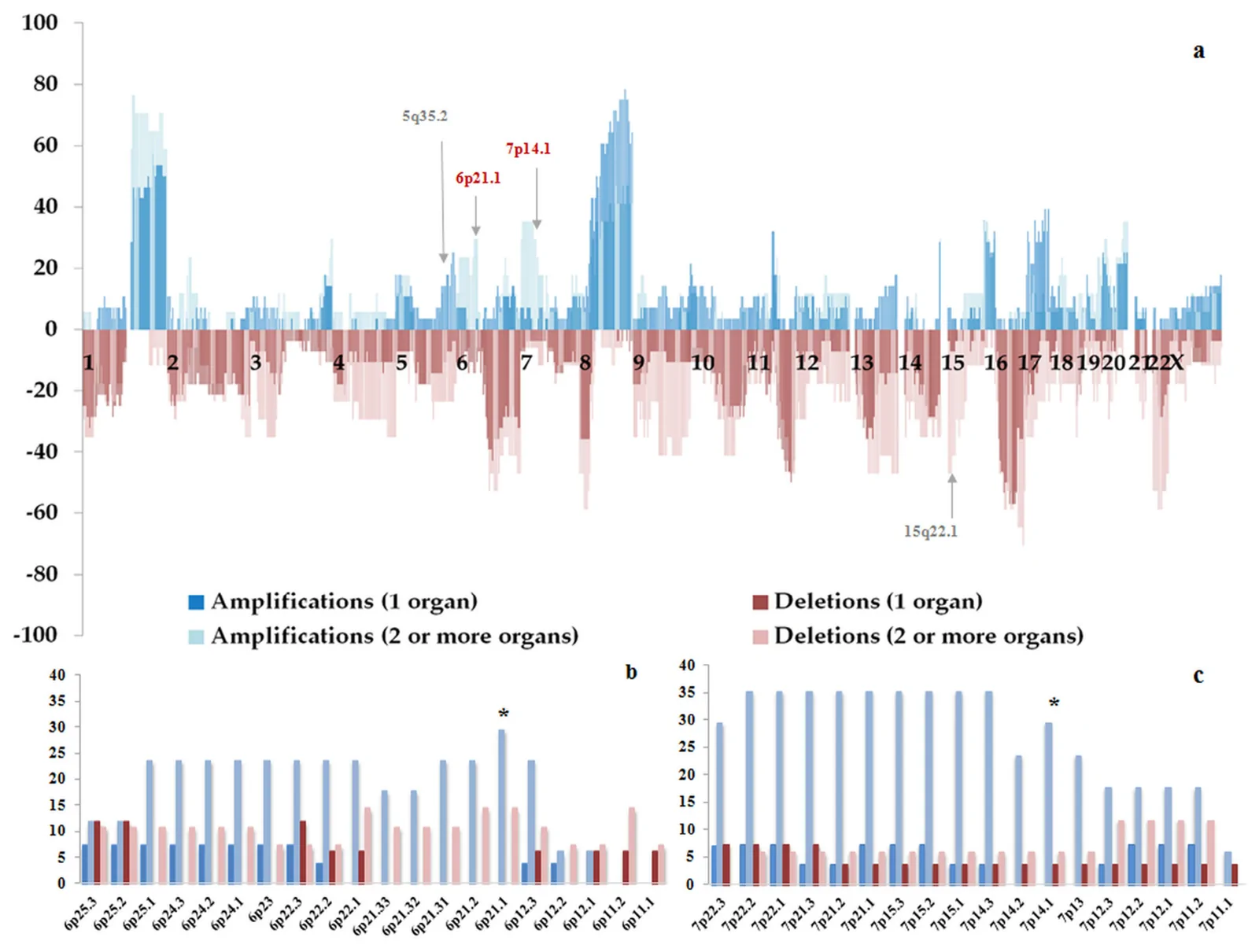
Frequency of CNAs in pretreatment tumors in patients with distant metastasis to a single organ (1 organ) and in patients with metastases to multiple organs (two or more organs). (**a**)–frequency of CNAs in the overall cohort of patients included in the study; (**b**)–frequency of CNAs in the 6p region in pretreatment tumors in patients with single-organ versus multiple-organ metastases; (**c**)–frequency of CNAs in the 7p region in pretreatment tumors in patients with single-organ versus multiple-organ metastases. Note: *—statistically significant difference (*p* < 0.05).

**Figure 2:**
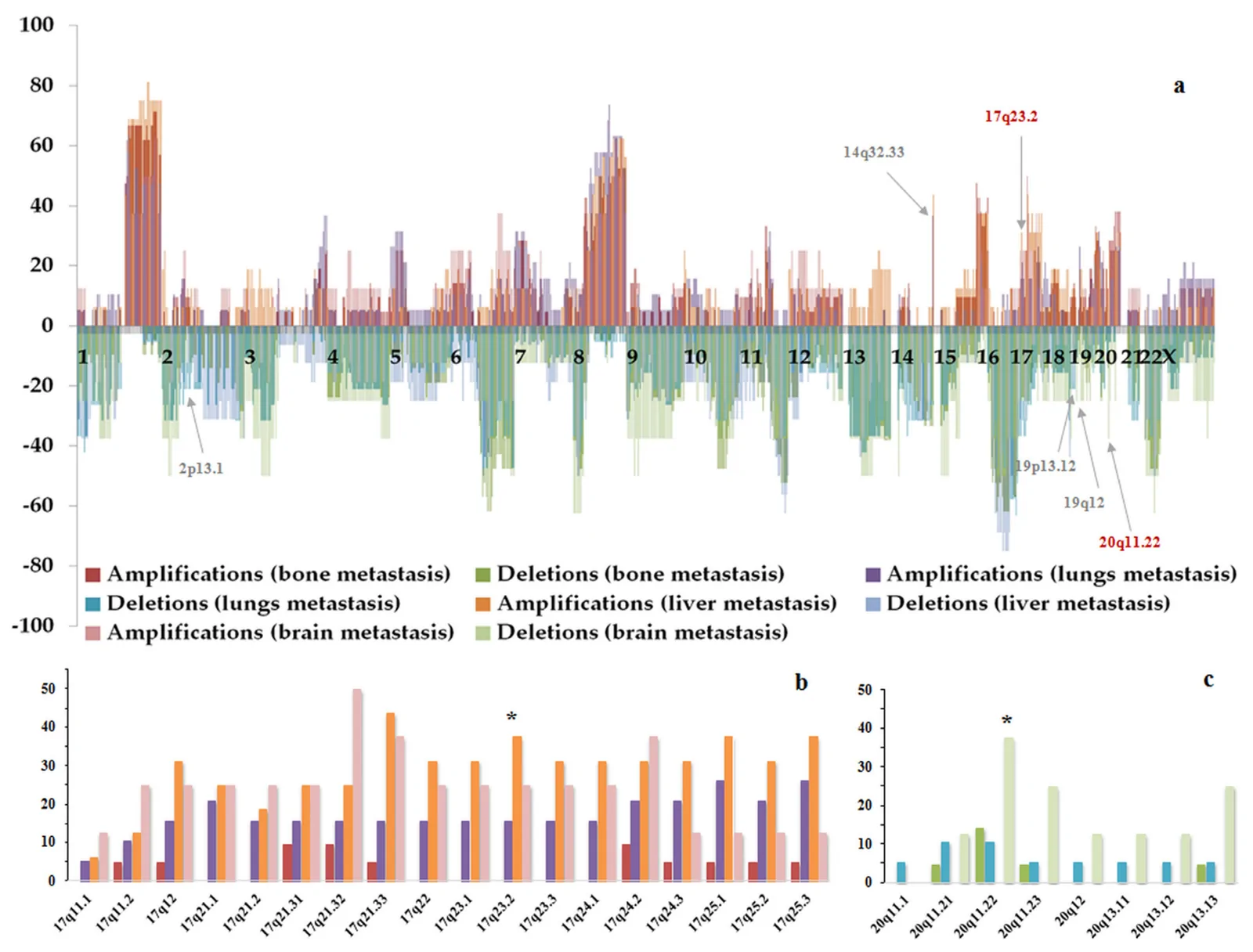
Frequency of CNA occurrence in pretreatment tumors according to organ-specific metastasis patterns. (**a**)–CNA frequencies in the overall cohort of patients included in the study, according to metastatic site; (**b**)–frequency of amplification in the 17q region in pretreatment tumors according to metastatic site; (**c**)–frequency of deletion in the 20q region in pretreatment tumors according to metastatic site. Note: No deletions were identified across the entire length of the long arm of chromosome 20 in cases of liver metastasis; additionally, no deletions were detected in the regions 20q13.2, 20q13.31, 20q13.32, and 20q13.33 regardless of the metastatic pattern; *—statistically significant difference (*p* < 0.05).

**Figure 3:**
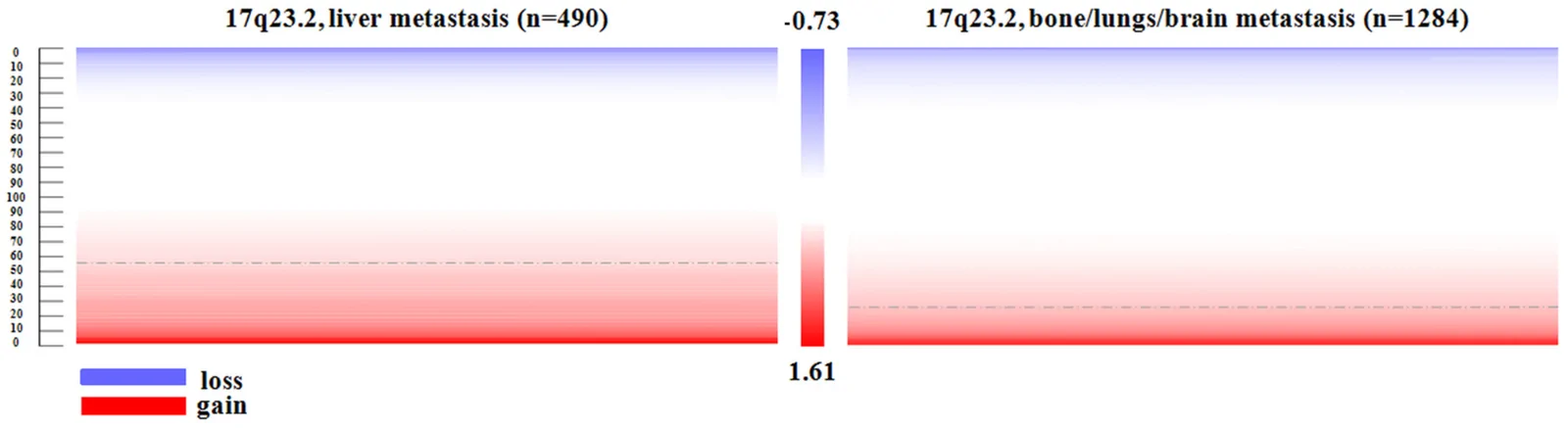
Frequency of amplifications at 17q23.2 in cases of liver metastasis and metastasis to other distant organs, according to data from the publicly available cBioPortal database.

**Figure 4:**
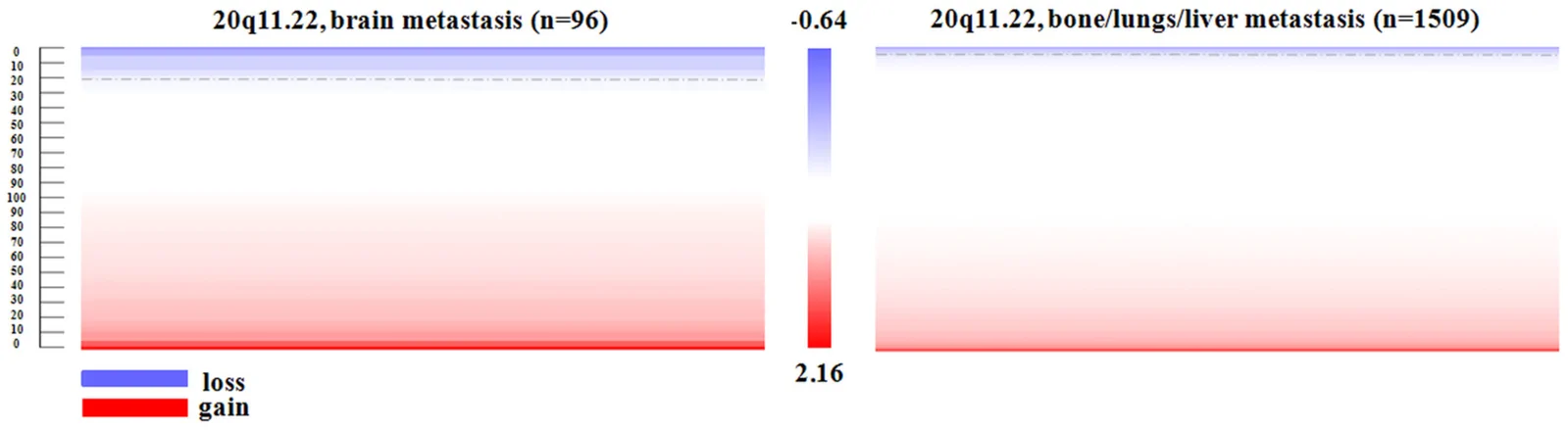
Frequency of deletion at 20q11.22 in cases of brain metastasis and metastases to other distant organs according to data from the publicly available cBioPortal database.

**Table 1:** Clinical and morphological characteristics of the patients with breast cancer included in the study.

Clinical and Morphological Parameter		Number of Patients (Absolute Number, %)
Age (years)	≤45	21 (42.0%)
	>45	29 (58.0%)
Menstrual status	Premenopausal	41 (82.0%)
	Postmenopausal	9 (18.0%)
Histological type	Invasive ductal carcinoma	36 (72.0%)
	Invasive lobular carcinoma	4 (8.0%)
	Medullary carcinoma	1 (2.0%)
	Other types	9 (18.0%)
Tumor size	T_1_	2 (4.0%)
	T_2_	36 (72.0%)
	T_3_	5 (10.0%)
	T_4_	7 (14.0%)
Lymphogenous metastasis	N_0_	10 (20.0%)
	N_1_	23 (46.0%)
	N_2_	5 (10.0%)
	N_3_	12 (24.0%)
Histological form	Unicentric	24 (48.0%)
	Multicentric	26 (52.0%)
NAC scheme	CAX	10 (20.0%)
	FAC/AC	28 (56.0%)
	Taxotere	10 (20.0%)
	CP	2 (4.0%)
Response to NAC	Progressive disease	4 (8.0%)
	Stable disease	20 (40.0%)
	Partial regression	26 (52.0%)
Average observation time for patients, months (M ± SE)		63.8 ± 1.2 (min–max:12–184)
Recurrence rate		8 (16.0%)

**Table 2:** Clinical and morphological characteristics of the patients with primary metastatic breast cancer included in the study.

Clinical and Morphological Parameter		Number of Patients (Absolute Number, %)
Age (years)	≤45	1 (25.0%)
	>45	3 (75.0%)
Menstrual status	Premenopausal	2 (50.0%)
	Postmenopausal	(50.0%)
Histological type	Invasive ductal carcinoma	2 (50.0%)
	Invasive lobular carcinoma	2 (50.0%)
Tumor size	T_3_	2 (50.0%)
	T_4_	2 (50.0%)
Lymphogenous metastasis	N_2_	2 (50.0%)
	N_3_	2 (50.0%)
Histological form	Unicentric	2 (50.0%)
	Multicentric	2 (50.0%)
NAC scheme	FAC/AC	4 (100%)
Response to NAC	Progressive disease	1 (25.0%)
	Stable disease	2 (50.0%)
	Partial regression	1(25.0%)
Average observation time for patients, months (M ± SE)		7.8 ± 0.3 (min–max:7–24)

**Table 4:** Status of CNA regions associated with organ-specific metastatic-site patterns in biopsy, surgical, and metastatic samples from patients with primary metastatic breast cancer according to organ-specific metastasis patterns.

Metastatic Site	CNA Regions	Case 1. Primary Metastatic BC, Bone Metastasis			Case 2. Primary Metastatic BC, Lung Metastasis			Case 3. Primary Metastatic BC, Liver Metastasis			Case 4. Primary Metastatic BC, Brain Metastasis		
		B	S	M	B	S	M	B	S	M	B	S	M
Bones (n = 21)	14q32.33 (gain)	gain	n	gain	n	gain	n	loss	loss	n	n	n	n
Lungs (n = 19)	2p13.1 (loss)	n	n	n	loss	loss	loss	gain	n	n	gain	n	n
	19p13.12 (loss)	gain	loss	loss	gain	n	gain	n	n	n	n	n	gain
Liver (n = 16)	**17q23.2 (gain)**	loss	loss	loss	n	gain	gain	gain	n	gain	n	n	n
Brain (n = 8)	19q12 (loss)	n	gain	gain	gain	n	gain	n	gain	n	loss	loss	loss
	**20q11.22 (loss)**	gain	n	n	n	n	n	gain	n	n	loss	n	loss

Notes: B—biopsy; S—surgical specimen; M—metastasis. Amplifications highlighted in bold indicate loci for which the frequency of amplification in liver metastases was statistically significant after Bonferroni correction compared with that in metastases to other organs. Deletions highlighted in bold indicate loci for which the frequency of deletion in brain metastases was statistically significantly higher after Bonferroni correction than that in metastases to other organs. The status of these loci in the corresponding biopsy, surgical, and metastatic samples is shown in red.

**Table 5:** Involvement of CNA regions associated with organ-specific metastatic-site patterns in cellular signaling pathways.

Metastatic Site	CNA Regions	Signaling Pathways
Bones	14q32.33 (gain)	PI3K/AKT/mTOR, Notch Signaling Pathway, DLK1-DIO3 imprinted cluster and MicroRNA (miRNA) signaling
Lungs	2p13.1 (loss)	DCTN1 and Retrograde Axonal Transport, CYP26B1 and Retinoic Acid (RA) Degradation, DOK1 and Insulin/MAPK Signaling.
	19p13.12 (loss)	NOTCH3 Signaling Pathway
Liver	17q23.2 (gain)	TGFβ/BMP-Smad signaling pathway
Brain	19q12 (loss)	Cell Cycle Pathway, URI1 Oncogenic Signaling
	20q11.22 (loss)	TGF-β/SMAD, Notch Signaling Pathway, Wntβ-catenin signaling pathway

**Table 6:** CNA status in biopsy, surgical, and metastatic specimens from patients with primary metastatic breast cancer according to organ-specific metastasis under neoadjuvant chemotherapy.

Metastatic Site	CNA Regions	Case 1. Primary Metastatic BC, Bone Metastasis			Case 2. Primary Metastatic BC, Lung Metastasis			Case 3. Primary Metastatic BC, Liver Metastasis			Case 4. Primary Metastatic BC, Brain Metastasis		
		B	S	M	B	S	M	B	S	M	B	S	M
Bones (n = 21)	**17q23.1 (gain)**	n	gain	gain	n	n	n	loss	loss	loss	gain	n	n
	2p12 (loss)	n	n	n	gain	n	n	gain	loss	loss	gain	n	n
	3p11.1 (loss)	n	loss	loss	n	n	loss	n	loss	n	n	n	n
Lungs (n = 19)	2p16.3 (gain)	n	n	n	n	gain	gain	loss	n	n	gain	n	n
	6q15 (gain)	n	n	n	n	gain	gain	loss	gain	gain	gain	n	gain
	Xq11.2 (loss)	n	n	n	n	gain	gain	n	loss	loss	gain	n	gain
	Xq21.31 (loss)	n	n	n	n	gain	gain	n	loss	loss	loss	n	loss
Liver (n = 16)	**7p12.3 (gain)**	n	n	gain	loss	n	loss	n	gain	gain	gain	n	n
	2p25.1 (gain)	n	n	n	n	n	n	gain	n	n	gain	n	gain
	1p12 (loss)	gain	n	n	n	n	n	loss	loss	loss	loss	n	n
	10q26.3 (loss)	n	n	n	n	n	n	loss	n	n	n	n	n
Brain (n = 8)	17q24.2 (gain)	n	gain	gain	n	n	n	loss	loss	loss	gain	n	n

Note: B—biopsy; S—surgical specimen; M—metastasis. Amplifications highlighted in bold indicate loci for which a statistically significant change in frequency (after Bonferroni correction) pre- vs. post- NAC was observed in metastasis to specific target organs. The statuses of these loci in the sample variants (biopsy, surgical, and metastatic samples) for the corresponding type of metastasis are shown in red.

## Data Availability

The datasets presented in this article are not readily available because the data are part of an ongoing study and due to technical and legal limitations. The data that support the findings of this study are available from the corresponding author upon reasonable request.

## Supplementary Materials

The following supporting information can be downloaded at: https://www.mdpi.com/article/10.3390/diseases14100352/s1, Table S1: A simultaneous comparison of SNA across four groups (stratified by the target organ of metastasis).

## Abbreviations

The following abbreviations are used in this manuscript:

BC	breast cancer
CNA	copy number aberration
NAC	neoadjuvant chemotherapy
TNBC	triple-negative breast cancer
HER2	human epidermal growth factor receptor 2
WHO	World Health Organization
CBS	circular binary segmentation
